# Prevalence, Disparities, and Trends in Obesity and Severe Obesity Among Students in the School District of Philadelphia, Pennsylvania, 2006–2013

**DOI:** 10.5888/pcd12.150185

**Published:** 2015-08-20

**Authors:** Jessica M. Robbins, Giridhar Mallya, Amanda Wagner, James W. Buehler

**Affiliations:** Author Affiliations: Giridhar Mallya, Amanda Wagner, James W. Buehler, Philadelphia Department of Public Health, Philadelphia, Pennsylvania.

## Abstract

**Introduction:**

Recent analyses suggest that increases in rates of childhood obesity have plateaued nationally and may be decreasing among certain populations and communities, including Philadelphia, Pennsylvania. We examined 7 years of data, including 3 years not previously reported, to assess recent trends in major demographic groups.

**Methods:**

We analyzed nurse-measured data from the School District of Philadelphia for school years 2006–07 through 2012–13 to assess trends in obesity (body mass index [BMI] ≥95th percentile) and severe obesity (BMI ≥120% of the 95th percentile) among all children aged 5 to 18 years for whom measurements were recorded.

**Results:**

Over 7 school years, the prevalence of childhood obesity declined from 21.7% to 20.3% (*P* = .01); the prevalence of severe obesity declined from 8.5% to 7.3% (*P* < .001). Declines were larger among boys than among girls and among African Americans and Asians than among non-Hispanic whites and Hispanics. Over the final 3 years of study, the prevalence of obesity continued to decrease significantly among boys (including African Americans and Asians) but increased significantly among Hispanic girls and girls in grades kindergarten through 5. At the end of the study period, Hispanics had the highest prevalence of obesity among boys (25.9%) and girls (23.0%). The prevalence of severe obesity continued to trend downward in boys and decrease significantly among girls (including African American girls) but remained highest among Hispanic boys (10.1%) and African American girls (8.3%).

**Conclusion:**

The prevalence of obesity and severe obesity continued to decline among children in Philadelphia, but in some groups initial reductions were reversed in the later period. Further monitoring, community engagement, and targeted interventions are needed to address childhood obesity in urban communities.

## Introduction

Since the 1970s, the prevalence of childhood obesity in the United States has more than tripled, raising concerns for the health of children and the health of the US population (1,2). Children with obesity are at increased risk for type 2 diabetes, cardiovascular disease, sleep apnea, orthopedic problems, and psychosocial distress (3). In 2012, 16.9% of children aged 2 to 19 years were obese. African American and Hispanic children had the highest obesity prevalence (4).

However, recent data suggest that the prevalence of childhood obesity is plateauing and may be decreasing among certain populations and in particular communities (4,5). Most notably, the prevalence of obesity declined nationally by 40% among children aged 2 to 5 between National Health and Nutrition Examination Survey (NHANES) 2003–04 and NHANES 2011–12 (4). In that same period (2003–2012), reductions in obesity prevalence among school-aged children ranged from 1% to 13% in California; Mississippi; New Mexico; West Virginia; Kearney, Nebraska; New York City; and Philadelphia (5). Local and state declines were seen over a period of 6 or fewer years, so long-term trends are unclear. Moreover, there were generally larger reductions in obesity prevalence among white children than among racial/ethnic minority children, exacerbating existing disparities. (6,7).

In the context of these reports, we updated an earlier study of obesity among Philadelphia public school children from school year 2006–07 through school year 2009–10 (8). That earlier analysis found a 4.8% decline in the prevalence of obesity, with larger declines for African American boys and Hispanic girls. Our objective was to examine obesity trends among children in different grade categories (kindergarten [K]–5, 6–8, and 9–12) and by sex and racial/ethnic groups. We examined 3 additional years of height and weight data from the School District of Philadelphia and assessed trends both in the period since the earlier study and over the 7-year period from school years 2006–07 through 2012–13.

## Methods

The study population consisted of students whose heights and weights were measured in traditional (noncharter) K to 12 public schools operated by the School District of Philadelphia in the period from September 2006 through June 2013. During this period, the total number of students aged 5 to 18 attending such schools averaged 168,960, or 63% of the Philadelphia population aged 5 to 18 as enumerated in the 2010 Census (9). The racial/ethnic composition of the students resembled that of all children in Philadelphia (54% African American, 18% Hispanic, 22% non-Hispanic white, 6% Asian) (9), except that there were fewer non-Hispanic whites. The data source and analytic methods have been described in detail previously (8). Briefly, heights and weights were measured by school nurses in accordance with state regulations requiring these measurements be made annually. These data, with the students’ dates of birth, measurement dates, sex, and race/ethnicity, were made available (without other identifiers) for each school year from 2006–07 through 2012–13. Although the policy was to measure all students in all grades, measurements were not conducted on all children each year, primarily because of student absences and resource limitations. The data were therefore weighted in each school year to adjust for unequal probabilities of having height and weight assessed as associated with grade, sex, and race/ethnicity.

We calculated body mass index (BMI) in BMI percentiles by using age-specific and sex-specific growth charts from the Centers for Disease Control and Prevention (10). Obesity was defined as a BMI percentile of 95% or more, and severe obesity as a BMI percentile of 120% or more of the obesity threshold (11). Students who reported a pregnancy during the school year and students with recorded heights below 0.2 m or above 3.0 m or weights less than 0.5 kg or more than 300 kg were excluded from the analyses; these out-of-range heights and weights were considered data entry errors. We did not exclude other students with extreme BMI *z*-score values to avoid any potential for underestimating the prevalence of severe obesity. We examined race/ethnicity and grade patterns separately by sex.

Since the prior analysis, data sets for school years 2006–07 through 2009–10 were updated on the basis of current data on birth dates, race/ethnicity, and sex, and exclusion criteria were reapplied. This led to small changes in the total number of students included overall and by subgroup and small changes in obesity and severe obesity prevalence estimates for those years.

All significance tests were carried out in SAS version 9.2 (SAS Institute, Inc). We used generalized estimating equation methods (SAS’s PROC SURVEYLOGISTIC) to adjust for the possibility that observations might be correlated within schools as a result of school-level variations in measurement methods. Models were tested for the entire study population and for boys and girls separately and adjusted for race/ethnicity, age in years, and grade. Stratified analyses were also carried out for each sex for 3 grade categories and 5 racial/ethnic groups to assess time trends within groups. These analyses were carried out separately for the 3 periods: school years 2006–07 through 2009–10 (the initial study period), 2009–10 through 2012–13 (the follow-up study period), and 2006–07 through 2012–13 (the entire study period).

## Results

The number of enrolled K to 12 students in the School District of Philadelphia declined each year, from a high of 186,176 in the 2006–07 school year to 147,818 in the 2012–13 school year (Table 1); the number of students with valid height and weight measurements varied by school year, with a high of 122,448 in 2009–10 and a low of 88,798 in 2012–13 (Table 2). The proportion of students whose BMI status could be assessed reached a high of 73% in 2010–11 before declining to 60% in 2012–13.

**Table 1:** Demographic Characteristics of Students Aged 5 Through18 Years With Measured Weight, School District of Philadelphia, Pennsylvania, 2006–2013

Characteristic	School Year						
	2006–07	2007–08	2008–09	2009–10	2010–11	2011–12	2012–13
**All students, n**	186,176	180,082	175,110	172,572	165,204	155,756	147,818
**Boys**							
Total, n	96,135	92,943	90,395	88,937	85,235	80,419	76,172
Grade category^a^, n (%)							
K–5	43,444 (45.2)	42,511 (45.7)	42,033 (46.5)	42,086 (47.3)	40,515 (47.5)	40,076 (49.8)	38,689 (50.8)
6–8	22,516 (23.4)	21,150 (22.8)	19,943 (22.1)	19,277 (21.7)	18,454 (21.7)	17,440 (21.7)	16,119 (21.2)
9–12	29,747 (30.1)	28,890 (31.1)	28,014 (31.0)	27,156 (30.5)	25,850 (30.3)	22,448 (27.9)	21,191 (27.8)
Race/ethnicity, n (%)							
African American	60,188 (62.6)	57,392 (61.7)	55,187 (61.1)	53,204 (59.8)	49,172 (57.7)	44,893 (55.8)	41,269 (54.2)
Hispanic	15,934 (16.6)	15,922 (17.1)	15,650 (17.3)	15,716 (17.7)	15,491 (18.2)	14,916 (18.5)	14,172 (18.6)
Non-Hispanic white	13,467 (14.0)	12,908 (13.9)	12,529 (13.9)	12,227 (13.8)	12,066 (14.2)	11,616 (14.4)	11,101 (14.6)
Asian	5,414 (5.6)	5,370 (5.8)	5,427 (6.0)	5,554 (6.2)	5,741 (6.7)	5,693 (7.1)	5,661 (7.4)
Other	1,132 (1.2)	1,351 (1.5)	1,602 (1.8)	2,186 (2.5)	2,765 (3.2)	3,301 (4.1)	3,969 (5.2)
**Girls**							
Total, n	90,041	87,139	84,715	83,635	79,969	75,337	71,646
Grade category, n (%)^a^							
K–5	39,778 (44.2)	38,941 (44.7)	38,462 (45.4)	38,709 (46.3)	37,175 (46.5)	36,758 (48.8)	35,829 (50.0)
6–8	21,075 (23.4)	19,528 (22.4)	18,345 (21.7)	17,690 (21.2)	16,930 (21.2)	16,045 (21.3)	14,845 (20.7)
9–12	28,848 (32.0)	28,332 (32.5)	27,578 (32.6)	26,902 (32.2)	25,543 (31.9)	22,149 (29.4)	20,887 (29.2)
Race/ethnicity, n (%)							
African American	56,818 (63.1)	54,166 (62.2)	52,207 (61.6)	50,518 (60.4)	46,710 (58.4)	42,657 (56.6)	39,560 (55.2)
Hispanic	14,963 (16.6)	14,944 (17.1)	14,623 (17.3)	14,759 (17.6)	14,473 (18.1)	13,985 (18.6)	13,387 (18.7)
Non-Hispanic white	12,030 (13.4)	11,612 (13.3)	11,225 (13.3)	11,112 (13.3)	10,995 (13.7)	10,646 (14.1)	10,324 (14.4)
Asian	5,159 (5.7)	5,197 (6.0)	5,234 (6.2)	5,425 (6.5)	5,630 (7.0)	5,550 (7.4)	5,437 (7.6)
Other	1,071 (1.2)	1,220 (1.4)	1,426 (1.7)	1,821 (2.2)	2,161 (2.7)	2,499 (3.3)	2,938 (4.1)

Abbreviations: K, kindergarten.

a Values may not sum to total due to missing data on grade.

**Table 2:** Demographic Characteristics of Students Aged 5 Through 18 Years With Measured Heights and Weights, School District of Philadelphia, Pennsylvania, 2006–2013

Characteristic	School Year						
	2006–07	2007–08	2008–09	2009–10	2010–11	2011–12	2012–13
**All students, n**	114,909	120,165	121,852	122,448	120,165	96,172	88,798
**Boys**							
Total, n	58,802	61,218	62,053	62,422	61,149	48,889	45,292
Grade category^a^, n (%)							
K–5	29,763 (50.8)	30,984 (50.6)	31,376 (50.6)	32,240 (51.6)	31,390 (51.3)	26,121 (53.4)	23,809 (52.6)
6–8	14,796 (25.2)	14,676 (24.0)	13,981 (22.5)	14,303 (22.9)	13,794 (22.6)	11,161 (22.8)	10,316 (22.8)
9–12	14,044 (23.9)	15,352 (25.1)	16,539 (26.7)	15,691 (25.1)	15,732 (25.7)	11,451 (23.4)	11,116 (24.5)
Race/ethnicity, n (%)							
African American	34,769 (59.1)	36,505 (59.6)	36,990 (59.6)	36,690 (58.8)	33,834 (55.3)	25,826 (52.8)	22,397 (49.5)
Hispanic	10,065 (17.1)	10,347 (16.9)	10,545 (17.0)	10,548 (16.9)	10,845 (17.7)	8,724 (17.8)	8,522 (18.8)
Non-Hispanic white	9,520 (16.2)	9,439 (15.4)	9,213 (14.8)	9,048 (14.5)	9,512 (15.6)	8,113 (16.6)	7,900 (17.4)
Asian	3,670 (6.2)	3,964 (6.5)	4,111 (6.6)	4,467 (7.2)	4,835 (7.9)	4,081 (8.3)	3,976 (8.8)
Other	778 (1.3)	963 (1.6)	1,194 (1.9)	1,669 (2.7)	2,123 (3.5)	2,145 (4.4)	2,497 (5.5)
**Girls**							
Total, n	56,107	58,947	59,799	60,026	59,016	47,283	43,506
Grade category^a^, n (%)							
K–5	27,598 (49.2)	28,704 (48.7)	29,071 (48.6)	29,900 (49.8)	29,126 (49.4)	24,385 (51.6)	22,455 (51.6)
6–8	14,002 (25.0)	13,708 (23.5)	12,909 (21.6)	13,232 (22.0)	12,876 (21.8)	10,456 (22.1)	9,671 (22.2)
9–12	14,507 (25.9)	16,250 (27.6)	17,582 (29.4)	16,641 (27.7)	16,727 (28.3)	12,222 (25.8)	11,334 (26.1)
Race/ethnicity, n (%)							
African American	33,816 (60.0)	35,348 (60.0)	36,195 (60.5)	35,868 (59.8)	33,389 (56.6)	25,610 (54.2)	22,148 (50.9)
Hispanic	9,507 (16.9)	10,070 (17.1)	10,122 (16.9)	10,015 (16.7)	10,261 (17.4)	8,391 (17.7)	8,009 (18.4)
Non-Hispanic white	8,633 (15.3)	8,662 (14.7)	8,336 (13.9)	8,316 (13.9)	8,833 (15.0)	7,559 (16.0)	7,439 (17.1)
Asian	3,652 (6.5)	3,941 (6.7)	4,060 (6.8)	4,419 (7.4)	4,795 (8.1)	4,059 (8.6)	3,940 (9.1)
Other	755 (1.3)	926 (1.6)	1,086 (1.8)	1,408 (2.3)	1,738 (2.9)	1,664 (3.5)	1,970 (4.5)

Abbreviations: K, kindergarten.

a Values may not sum to total due to missing data on grade.

The majority of enrolled students and the majority with BMI assessments were African American, but the proportion of African American students declined over the 7 years. The percentage of students who were Hispanic, non-Hispanic white, or Asian rose slightly (Table 1, Table 2).

### Obesity

Over the full 7-year study period from 2006–07 through 2012–13, the prevalence of obesity among all children decreased from 21.7% to 20.3%, a relative decline of 6.3% (*P* = .01). Relative obesity reductions were larger in the first 4 years (4.6%) than in the last 3 years (1.8%).

Among boys, the prevalence of obesity declined from 21.9% to 20.1% over the 7-year period, a relative decline of 8.1% (*P* < .001, Table 3). The largest percentage declines were seen in grades K to 5 (8.8%) and among African Americans (11.3%) and Asians (18.8%). Every racial/ethnic group had a lower prevalence of obesity in 2009–10 than in 2006–07, but only African American and Asian boys had a significantly lower prevalence in 2012–13 than in 2009–10. Hispanic boys experienced the smallest decline from 2006–07 through 2012–13 (1.7%), which was nonsignificant; they also had the highest obesity prevalence throughout the 7-year period.

**Table 3:** Obesity^a^ Prevalence Among Students Aged 5 Through 18 Years With Measured Heights and Weights, by Demographic Characteristics, School District of Philadelphia, Pennsylvania, 2006–2013

Characteristic	School Year, %							% Change 2006–07 to 2009–10	*P* Value for Trend 2006–07 to 2009–10^b^	% Change 2009–10 to 2012–13	*P* Value for Trend 2009–10 to 2012–13^b^	% Change 2006–07 to 2012–13	*P* Value for Trend 2006–07 to 2012–13^b^
	2006–07	2007–08	2008–09	2009–10	2010–11	2011–12	2012–13						
**All students**	21.7	21.4	20.8	20.7	20.9	20.7	20.3	−4.6	<.001	−1.8	.46	−6.3	.01
**Boys**													
Total	21.9	21.4	20.5	20.6	20.9	20.5	20.1	−5.8	<.001	−2.4	.03	−8.1	<.001
Grade category													
K–5	21.0	20.7	19.4	19.5	19.9	19.5	19.2	−7.0	<.001	−1.9	.11	−8.8	<.001
6–8	23.7	23.0	22.5	23.0	23.3	22.4	22.1	−3.0	.04	−3.9	.06	−6.7	.04
9–12	21.7	21.3	20.7	20.5	20.7	20.8	20.2	−5.6	.002	−1.6	.73	−7.1	.07
Race/ethnicity													
African American	20.7	20.2	19.3	19.2	19.6	19.3	18.4	−7.4	<.001	−4.2	.04	−11.3	<.001
Hispanic	26.3	26.2	24.9	25.8	26.0	25.5	25.9	−2.2	.31	0.6	.80	−1.7	.77
Non-Hispanic white	22.4	21.9	21.3	21.1	21.0	20.3	20.8	−5.9	.02	−1.2	.47	−7.0	.03
Asian	20.2	19.3	18.4	18.5	18.3	17.6	16.4	−8.3	.04	−11.5	.02	−18.8	.001
Other	20.6	21.2	20.0	19.5	19.7	19.7	20.2	−5.1	.30	3.3	.70	−2.0	.71
**Girls**													
Total	21.4	21.4	21.0	20.7	20.9	20.9	20.5	−3.3	.07	−1.0	.40	−4.3	.75
Grade category													
K–5	19.9	20.1	19.4	19.2	19.6	20.0	19.5	−3.9	.02	1.9	.02	−2.0	.67
6–8	24.8	23.7	23.1	23.4	24.0	23.5	23.3	−5.7	.04	−0.5	.97	−6.1	.56
9–12	21.0	21.6	22.1	21.2	20.9	20.4	20.3	1.0	.35	−4.6	.42	−3.6	.52
Race/ethnicity													
African American	23.1	23.1	23.0	22.8	23.1	22.5	22.2	−1.2	.63	−2.6	.34	−3.8	.49
Hispanic	22.7	22.6	21.4	20.9	21.8	23.3	23.0	−7.8	.003	10.1	<.001	1.5	.20
Non-Hispanic white	17.5	17.8	17.3	17.5	17.2	17.3	17.7	−0.5	.60	1.5	.61	1.0	.93
Asian	9.5	9.2	9.4	9.0	9.1	9.0	8.8	−5.3	.48	−1.9	.68	−7.1	.32
Other	18.8	19.2	17.5	17.5	17.8	18.3	18.2	−7.0	.54	4.0	.59	−3.3	.94

Abbreviations: K, kindergarten.

a Obesity was defined as a body mass index ≥95th percentile, according to Centers for Disease Control and Prevention growth charts (10). Data were weighted for nonresponse so that the measured population would more accurately represent the entire school population.

b Calculated using Wald χ^2^ type 3 analysis of effects. All tests were controlled for other variables shown in the table and single year of age.

Among girls, the prevalence of obesity trended downward, from 21.4% to 20.5% over the 7-year period, a relative decline of 4.3% (*P* = .75, Table 3). The decline was larger in the first 4 years (3.3%) than in the final 3 years (1.0%). None of the subgroups experienced significant reductions in obesity over the 7-year period. Overall, the largest reductions were seen in grades 6 to 8 (6.1%) and among African Americans (3.8%) and Asians (7.1%). There was a small but statistically significant increase (from 19.2% to 19.5%) among girls in grades K to 5. Among Hispanic girls, the prevalence of obesity initially declined from 22.7% to 20.9% from 2006–07 through 2009–10 but then increased significantly to 23.0% by school year 2012–13, representing the highest prevalence among girls in all racial/ethnic groups.

The prevalence of obesity was higher among boys than girls in 2006–07 (21.9% vs 21.4%) but was equal or higher among girls than boys in each subsequent school year.

### Severe obesity

The prevalence of severe obesity declined significantly for all children (8.5% to 7.3%, a relative decline of 13.9%), for boys (8.9% to 7.5%) and for girls (8.1% to 7.2%) over the 7-year period (Table 4); declines among girls continued over the last 3 years. The highest prevalence was in grades 6 to 8 for both boys and girls. The patterns by race/ethnicity were similar to those for obesity, and the largest reductions were among African Americans and Asians. Declines continued in all racial/ethnic groups, except for Hispanics, over the last 3 years. Notably, African American boys and girls experienced significant declines in the prevalence of severe obesity of 18.8% and 8.8% respectively over the 7-year period; most of the reduction for girls occurred in the final 3 years. Hispanic boys and girls experienced nonsignificant increases in the prevalence of severe obesity over the final 3 years and nonsignificant decreases (6.5% for boys and 7.5% for girls) over the entire study period. Boys had rates of severe obesity slightly higher than or equal to girls in all but 1 year.

**Table 4:** Severe Obesity^a^ Prevalence, Students Aged 5 through 18 Years With Measured Heights and Weights, by Demographic Characteristics, School District of Philadelphia, 2006–2013

Characteristic	School Year, %							% Change 2006–07 to 2009–10	*P* Value for Trend 2006–07 to 2009–10^b^	% Change 2009–10 to 2012–13	*P* Value for Trend 2009–10 to 2012–13^b^	% Change 2006–07 to 2012–13	*P* Value for Trend 2006–07 to 2012–13^b^
	2006–07	2007–08	2008–09	2009–10	2010–11	2011–12	2012–13						
**All students**	8.5	8.4	7.9	7.8	7.9	7.7	7.3	−8.2	<.001	−6.2	.01	−13.9	<.001
**Boys**													
Total	8.9	8.6	7.9	7.8	7.9	7.9	7.5	−11.8	<.001	−5.0	.09	−16.2	<.001
Grade category													
K–5	8.0	7.7	7.0	6.9	6.9	6.8	6.7	−13.4	<.001	−2.8	.30	−15.8	<.001
6–8	10.0	9.7	8.8	9.2	9.2	9.1	8.5	−7.8	.002	−7.6	.17	−14.8	.005
9–12	9.3	9.0	8.6	8.3	8.4	8.8	7.9	−11.4	.01	−4.1	.76	−15.0	.02
Race/ethnicity													
African American	8.8	8.5	7.8	7.6	7.8	7.9	7.2	−14.1	<.001	−5.4	.22	−18.8	<.001
Hispanic	10.8	10.4	9.6	9.9	9.9	9.8	10.1	−8.1	.02	1.8	.54	−6.5	.32
Non-Hispanic white	8.2	8.3	7.8	7.7	7.4	7.4	7.1	−5.7	.17	−8.2	.15	−13.4	.009
Asian	5.7	5.2	4.9	5.3	4.6	4.7	3.7	−7.1	.41	−30.0	<.001	−35.0	<.001
Other	8.5	7.1	6.7	6.3	6.1	6.6	7.3	−26.4	.06	16.2	.22	−14.5	.66
**Girls**													
Total	8.1	8.2	7.9	7.8	8.0	7.5	7.2	−4.0	.11	−7.5	.03	−11.2	.02
Grade category													
K–5	6.7	6.8	6.5	6.6	6.5	6.4	6.1	−2.1	.50	−6.7	.16	−8.6	.14
6–8	10.2	9.9	9.3	9.2	9.7	9.0	8.6	−10.3	.004	−6.0	.27	−15.7	.02
9–12	8.5	9.0	8.9	8.7	8.9	8.3	8.1	1.7	.48	−6.7	.23	−5.2	.75
Race/ethnicity													
African American	9.1	9.4	9.0	9.0	9.3	8.6	8.3	−0.8	.44	−8.0	.01	−8.8	.05
Hispanic	8.3	8.1	7.7	7.4	8.2	7.9	7.7	−11.6	.02	4.5	.53	−7.5	.49
Non-Hispanic white	6.0	5.8	6.0	6.0	5.7	5.5	5.8	0.3	.81	−3.5	.51	−3.2	.55
Asian	2.1	2.3	2.3	2.1	2.1	2.3	1.8	−2.4	.87	−14.0	.43	−16.1	.48
Other	6.2	6.9	5.5	5.3	5.8	5.6	5.3	−13.6	.21	−0.4	.83	−13.9	.29

Abbreviations: K, kindergarten.

a Severe obesity was defined as a body mass index ≥120% of the threshold for obesity, based on the recommendation of Flegal et al (11). Data were weighted for nonresponse so that the measured population would more accurately represent the entire school population.

b Calculated using Wald χ^2^ type 3 analysis of effects. All tests were controlled for other variables shown in the table and single year of age.

## Discussion

From school years 2006–07 through 2012–13, the prevalence of childhood obesity and severe obesity among Philadelphia school children declined by 6.3% and 13.9, respectively. Reductions were larger among boys than girls, among African Americans and Asians than among whites and Hispanics, and in the first 4 years than in the final 3 years. After initial declines, the prevalence of obesity increased significantly among Hispanic girls and girls in grades K to 5 over the final 3 years.

These findings in Philadelphia, the fifth largest US city and the poorest of the 10 largest cities in the nation, have similarities to and are different from findings in the nation as a whole and other communities. There were significant decreases in obesity between NHANES 2003–04 and NHANES 2010–11 only among 2- to 5-year-olds (4). Since NHANES 1999–2000, obesity generally increased among 6- to 19-year-olds except that levels plateaued among 6- to 11-year-olds boys between 2006–07 and 2011–12 (12). Since NHANES 1999–2000, the prevalence of obesity increased significantly for African American boys and Hispanic girls but remained stable over the last 2 years of NHANES data. Similar to Philadelphia data, NHANES data showed that Hispanics had the highest prevalence of obesity among both boys and girls.

Other communities across the country have recently reported reductions in obesity rates among school-aged children ranging from 1% to 13% (5). Most of these analyses focused on children in elementary or middle school and demonstrated reductions among a general population of children. Philadelphia is the only community to have reported larger reductions in obesity prevalence among certain racial/ethnic minority children than among white children. In New York City, the prevalence of obesity and severe obesity among children in grades K to 8 declined by 5.5% and 9.5%, respectively, from school years 2006–07 to 2010–11 (13). Decreases were largest among whites and smallest among African Americans, but all were significant.

Although our study did not explore factors associated with improvements in weight status, extensive school-based and community-based initiatives may have played a role. As noted previously (8), the School District of Philadelphia enacted a series of reforms in the 1990s and 2000s, including nutrition education in approximately 200 schools with high rates of poverty, greater access to free or reduced-price meals through the National School Lunch Program and the School Breakfast Program, comprehensive nutrition standards for foods offered in cafeterias and vending machines and at fundraisers, and shifts toward healthier items (low-fat milk) and cooking practices (removal of kitchen fryers). Since 2010, the Philadelphia Department of Public Health and its partners have implemented Get Healthy Philly, a community-wide effort to implement policy and systems changes for improved nutrition and physical activity. Key activities that may have affected the weight status of school-aged children were creation of Wellness Councils in 170 public schools serving 100,000 students to ensure that health-promoting policies — such as healthy food fundraisers and classroom movement breaks — are put into practice (14); citywide food and fitness standards for 300 after-school sites serving 20,000 low-income children (14); 650 healthy corner stores offering more fruits, vegetables, and low-fat milk (15); *Safe Routes to School* bike and pedestrian education affecting 50,000 second-graders and fifth-graders (14); and a mass-media campaign highlighting links between sugary drink consumption, weight gain, and diabetes in children (16). Between 2007 and 2013, soda consumption among Philadelphia teens declined by 24%; in contrast, fruit and vegetable consumption, physical activity, and screen time among Philadelphia teens did not change over this same time period (17). Along with local efforts, state and national policies may have also played a role in the childhood obesity declines seen in Philadelphia (6,7).

Despite reductions in obesity prevalence among children overall and among certain racial/ethnic minorities, Hispanic children are lagging, particularly Hispanic girls. During the study period, Hispanic girls experienced a significant drop in the prevalence of obesity, from 22.7% to 20.9% (2006–07 to 2009–10) and then a significant increase to 23.0% by 2012–13. Hispanic children may face unique challenges with nutrition and physical activity. Research at the national level suggests that Hispanic children are more likely to attend schools that offer fast food in cafeterias (18) and unhealthy foods and beverages in school stores (19) than African American children are. Spanish-language media serve a rapidly growing market that food and beverage companies have explicitly targeted (20,21), resulting in larger increases in advertising expenditures for sugary cereals (22) and a larger percentage of advertisements from companies that have not adopted voluntary advertising standards for children compared with advertisers in English-language media (23). Moreover, advertisements from fast-food companies appearing on Spanish-language outlets are more likely to emphasize their support for community initiatives, emphasizing the message that they value Hispanics (24). Acculturation, which was not measured in our study, has mixed effects on physical activity among Hispanics but is associated with greater consumption of sugar and fast food (25–27). In Philadelphia, the highest levels of sugary drink intake are among Hispanic girls (17). On the basis of self-report, African American girls in Philadelphia engage in the least amount of physical activity and the most television viewing (17), but accelerometer-based measures reveal that Hispanic middle-school children in the city are significantly less likely than African American children to get 60 minutes of moderate to vigorous physical activity per day (28). Across racial/ethnic groups, girls were 5 times less likely than boys to meet physical activity targets. Finally, many local obesity prevention initiatives, such as the Food Fit Philly sugary drinks media campaign, have focused on African American children (16).

Data on nationality of origin, immigration status, and acculturation were not available for Hispanic school children. In Philadelphia as whole, the nationality of origin for Hispanics shifted from 67% Puerto Rican and 8% Mexican in 2005 to 66% Puerto Rican and 10% Mexican in 2013 (29). This may understate the changes in the local Hispanic population, because recent immigrants are more frequently uncounted in Census Bureau surveys than earlier immigrants or US-born residents (30). Further research and interventions prioritizing Hispanic children and girls are needed, nationally and in Philadelphia. Carrying out analyses by race/ethnicity separately by sex is an important strength of this study. Differing patterns by sex are consistently seen (1), and failing to stratify by sex paints an inaccurate picture of the racial/ethnic differences among boys and girls.

The Philadelphia Department of Public Health has begun to integrate these findings into its planning for future obesity prevention efforts: convening Hispanic-serving and girl-focused health and social service organizations to assess current obesity prevention practices, asking all organizations with chronic disease prevention contracts to specify how they will tailor interventions to reach girls and Hispanic children more effectively, more consistently incorporating culturally relevant Spanish-language messaging into nutrition and physical activity media campaigns, and pursuing policy strategies that may be particularly effective for these groups, such as minute-based physical education requirements in public schools and food and fitness standards for childcare providers.

Our study had several limitations. First, techniques and equipment used by school nurses to measure height and weight could not be assessed and may not have been consistent between schools or over time. Second, data were cross-sectional; individual-level changes in weight status were not assessed. A separate evaluation showed longitudinal reductions in BMI percentile in Philadelphia school children from school years 2010–11 to 2011–12 (31). Third, the population size decreased significantly from the first year (2006–07) to the last year (2012–13) of the study, but it remained large (n = 88,798). There were substantial shifts in enrollment over the study period, from traditional public schools to charter schools. In addition, the proportion of all school children that was assessed increased initially and then decreased to a baseline of approximately 60%. The decrease in the percentage of children assessed in 2012–13 may be due to the elimination of approximately 100 school nurse positions during that school year. Fourth, the population changed demographically over time; the percentage of African Americans decreased, and the percentages of Hispanics, non-Hispanic whites, and Asians increased. These changes likely had mixed effects on overall obesity prevalence. To minimize bias, we adjusted for demographic covariates in multivariable trend analyses; however, unmeasured confounders related to charter school shifts may have affected our results. Finally, we were not able to evaluate the underlying reasons for changes in weight status in this population.

This study of public school children in Philadelphia demonstrated significant reductions in the prevalence of obesity and severe obesity over a 7-year period. These declines were generally larger and more consistent than those seen in the United States as a whole, and the greater declines among African Americans and Asians are unique among communities reporting reductions in the prevalence of childhood obesity. With lesser gains among girls, reversals among Hispanic girls, and 1 in 5 children remaining obese, additional action is needed to improve nutrition and physical activity among youth in Philadelphia and across the United States.
